# Private equity investment in Europe’s primary care sector—a call for research and policy action

**DOI:** 10.1093/eurpub/ckad061

**Published:** 2023-04-13

**Authors:** Bernd Rechel, Florian Tille, Peter Groenewegen, Rob Timans, Giovanni Fattore, Katja Rohrer-Herold, Dheepa Rajan, Sophie Lopes

**Affiliations:** European Observatory on Health Systems and Policies, London School of Hygiene & Tropical Medicine, London, UK; European Observatory on Health Systems and Policies, London School of Hygiene & Tropical Medicine, London, UK; Netherlands Institute for Health Services Research (NIVEL), Utrecht, The Netherlands; Netherlands Institute for Health Services Research (NIVEL), Utrecht, The Netherlands; Università Bocconi, Milano, Italy; World Health Organization, Geneva, Switzerland; European Observatory on Health Systems and Policies, Brussels, Belgium; Caisse nationale de l'assurance maladie (CNAM), Paris, France

Corporate investment by private equity funds in primary care in Europe is a growing challenge but has so far received only scant attention. This is surprising, as it is beginning to change the healthcare landscape in many European countries.

In Germany, private equity investments in ambulatory health care centres [German: Medizinische Versorgungszentren (MVZ)] have increased significantly in recent years. In 2020, about 750 of overall 3800 ambulatory health care centres were estimated to be in the hands of private equity funds, despite regulations aimed at preventing this type of ownership.^1^ However, robust data on the ownership situation do not exist, as there are no legal requirements for disclosures and many investors are located in tax havens.

In Ireland, global investors have entered the market for newly built primary care centres, which are then leased by the Health Service Executive over 25 years, resulting in stable revenue streams and potentially higher costs to the public sector than in traditional forms of procurement.^2^

In the UK, one of the biggest general practitioner (GP) practice operators passed into the hands of the US health insurance group Centene Corporation in 2021. In 2022, a covert BBC Panorama investigation at one of the 51 surgeries found that physician associates (medically trained generalist health professionals who work alongside doctors) were used without adequate supervision by GPs, because they were ‘cheaper’ than GPs.^3^

In Sweden, a primary care reform in 2010 led to a rapid growth of private providers, accounting now for approximately 40% of the country’s 1200 primary care centres. About a third of the private-for-profit practices are owned by international private equity firms.^4^

In the Netherlands, commercial GP chains have emerged, some of which operate-for-profit and are owned by private equity firms. Our analysis of data on GP registrations and general practices suggests that the number of commercially run practices in 2022 was somewhere between 45 and 230 practices, out of a total of 4874 practices registered.

As these examples from across Europe illustrate, the scale of private equity investment in Europe’s primary care sector varies considerably between countries, but it seems to be increasing in many. The short investment and resale horizon of private equity funds and their tendency to consolidate practices into larger chains or consortia are of particular concern and set this type of ownership apart from primary care practices owned by physicians themselves or other owners (e.g. hospitals). Yet, information on the long-term impact of private equity investments, such as on equity, access to care, competition, the long-term economic prospects of providers (both those owned by private equity investors and other practices), data protection and health care costs is generally missing. In a qualitative study among physicians in Germany, participants also voiced concerns about diminished medical and managerial autonomy and the threat of declining quality of care due to the pursuit of more profitable treatment options.^5^

There are also potential benefits of private equity investments to be had, such as providing needed capital or improving efficiency. GP chains run by commercial investors might offer physicians the possibility of being employed, working part-time and working as part of a team. However, these potential benefits could also be had with different types of (private or public) ownership.

Political opposition to private equity involvements seems to be growing. In January 2023, the German Medical Chamber published a strategy paper on how to limit funding possibilities into primary care for investors without a medical background and the Federal Minister of Health, Karl Lauterbach, announced in late 2022 that a law prohibiting private equity acquisitions of ambulatory health care centres would be forthcoming in 2023. In the Netherlands, an investigation by the Inspectorate Health Care and Youth and the Dutch Health Care Authority into ‘innovative chains of GP care’ is currently ongoing.

We believe there is a strong need for research into the impact of corporate investment in primary care on patients, the health professionals working in these practices and primary care providers in the vicinity. Moreover, there is a strong case for more transparency on purchases and ownership structures and strengthened regulatory oversight to protect the interests of patients, health workers and the public, including public funding for health. More research on the impact of private equity investments on health and the health care landscape in Europe could provide pointers to where policy attention is most urgently needed.

## Data Availability

No new data were generated writing this editorial.
